# Supplementary material for: The effects of gluteal stretching vs. Lightback® on hip rotation range of motion and posterior chain flexibility in healthy subjects: a cross-over clinical trial
Source: PeerJ. 2025 Feb 5;13:e18523. doi: 10.7717/peerj.18523 (PMC11806910; doi:10.7717/peerj.18523)
Supplement: Supplemental Information 3 [file peerj-13-18523-s003.pdf]

Trial registered on ANZCTR

**Registration number**	ACTRN12624000300572
**Ethics application status**	Approved
**Date submitted**	8/11/2023
**Date registered**	21/03/2024
**Date last updated**	21/03/2024
**Date data sharing statement initially provided**	21/03/2024
**Type of registration**	Prospectively registered

Titles & IDs

**Public title**	The Effects of Gluteal Stretching vs LightBack® on Hip Mobility in Healthy Subjects: A Cross-Over Clinical Trial.
**Scientific title**	The Effects of Gluteal Stretching vs LightBack® on Passive Hip External Rotation in Healthy Subjects: A Cross-Over Clinical Trial.
**Secondary ID [1]**	Nil known
**Universal Trial Number (UTN)**	
**Trial acronym**	
**Linked study record**	

Health condition(s) or problem(s) studied:

decreased hip range of motion

Condition category	Condition code
Physical Medicine / Rehabilitation	Physiotherapy
Musculoskeletal	Normal musculoskeletal and cartilage development and function

Intervention/exposure

**Study type**	Interventional
**Description of intervention(s) / exposure**	One physiotherapist conducts one-on-one sessions with the patient, gradually increasing stretching until the patient reaches the maximum sensation without pain. Each stretching session lasts for 5 minutes, with a one-week washout period between treatments. Checklists were employed to assess patient interventions.<br><br>Two stretching techniques will be applied, one on each leg of the subject. The classic gluteal stretch will serve as the control group, while the stretching using the LightBack device will be the experimental group.<br><br>Experimental Group:<br>The LightBack® machine involves an anterior-posterior (A-P) mobilization of the femur, inducing a stretch of the posterior hip capsule and gluteal muscles. The subject will sit on the machine, bringing the knee towards the chest until it reaches 90° of hip flexion. When the subject feels a stretching sensation without pain, they will hold the position for 10 seconds. Subsequently, they will return to the initial relaxed position for 5 seconds and repeat the sequence 6 times for a total application time of one minute. It's a self-passive mobilization.
**Intervention code [1]**	Prevention
**Intervention code [2]**	Rehabilitation
**Comparator / control treatment**	One physiotherapist conducts one-on-one sessions with the patient, gradually increasing stretching until the patient reaches the maximum sensation without pain. Each stretching session lasts for 5 minutes, with a one-week washout period between treatments. Checklists were employed to assess patient interventions.<br><br>Control Group: Active control with the other limb-leg<br>On another day, the subject will perform a gluteal stretch on the other leg. This will be a passive type 1 stretch where the subject, in a supine position, will bring the leg to 90° of hip flexion and external hip rotation with the assistance of the other leg until they feel a stretching sensation. The stretch will have an application time of 30 seconds, and two repetitions will be performed for a total application time of 1 minute. 30 seconds rest between repetitions.
**Control group**	Active

Outcomes

**Primary outcome [1]**	Passive Hip External rotation range of motion
**Assessment method [1]**	Assessed by digital inclinometer in degree
Timepoint [1]	Baseline and Immediately after the treatment
**Secondary outcome [1]**	Active Knee Extension Test (AKE)
**Assessment method [1]**	Assessed by digital inclinometer in degree
Timepoint [1]	Baseline and Immediately after the treatment
**Secondary outcome [2]**	Active Straight Leg Raise (aSLR)
**Assessment method [2]**	Assessed by digital inclinometer in degree
Timepoint [2]	Baseline and Immediately after the treatment
**Secondary outcome [3]**	Passive Hip Internal Rotation range of motion
**Assessment method [3]**	Assessed by digital inclinometer in degree
Timepoint [3]	Baseline and Immediately after the treatment
**Secondary outcome [4]**	Active Hip Internal Rotation range of motion
**Assessment method [4]**	Assessed by digital inclinometer in degree
Timepoint [4]	Baseline and Immediately after the treatment
**Secondary outcome [5]**	Active Hip External Rotation range of motion
**Assessment method [5]**	Assessed by digital inclinometer in degree
Timepoint [5]	Baseline and Immediately after the treatment

Eligibility

**Key inclusion criteria**	- Healthy physically active participants<br>- Maintained a training regimen of at least two days per week
**Minimum age**	18 Years
**Maximum age**	30 Years
**Sex**	Both males and females
**Can healthy volunteers participate?**	Yes
**Key exclusion criteria**	- Exclusion criteria were individuals with a history of musculoskeletal lower limb or lumbopelvic conditions within the last five years, as well as those with neuromuscular, rheumatic, cardiovascular or neurological diseases, and those who had undergone previous surgical interventions or experienced fractures in the lower extremities or abdominal region.<br>- To be pregnant<br>- Allergic to a material in the machine<br>- To experience any type of pain during the procedure

Study design

**Purpose of the study**	Prevention
**Allocation to intervention**	Randomised controlled trial
**Procedure for enrolling a subject and allocating the treatment (allocation concealment procedures)**	central randomisation by computer
**Methods used to generate the sequence in which subjects will be randomised (sequence generation)**	Simple randomisation using a randomisation table created by computer software
**Masking / blinding**	Blinded (masking used)
**Who is / are masked / blinded?**	
	The people assessing the outcomes<br>The people analysing the results/data
**Intervention assignment**	Other
**Other design features**	Participants will be randomly allocated to receive the intervention stretch on one leg and the comparator stretch will then be applied to the opposite leg.
**Phase**	Not Applicable
**Type of endpoint/s**	Efficacy
**Statistical methods / analysis**	The statistical analysis was carried out using IBM SPSS Statistics version 29.0 software for Windows (IBM, Armonk, NY, USA). To assess data distribution, the Kolmogorov-Smirnov test and histogram examination were employed. For parametric variables ($p > 0.05$), descriptive statistics including mean and standard deviation were presented, while for non-parametric variables ($p < 0.05$), median and interquartile range were reported.<br>To compare the baseline characteristics of the two groups, either an independent t-test or Mann-Whitney U test was performed, taking into account assumptions of homoskedasticity and sphericity. If these assumptions were met, a two-way analysis of variance (ANOVA) with a 2 × 2 design was conducted. The effect size was assessed using partial eta squared (?2p), with values of 0.01 interpreted as small, 0.06 as medium, and 0.14 as large. A 95% confidence interval was set to all analyses.
